# Bimodal moment-by-moment coupling in perceptual multistability

**DOI:** 10.1167/jov.24.5.16

**Published:** 2024-05-31

**Authors:** Jan Grenzebach, Thomas G. G. Wegner, Wolfgang Einhäuser, Alexandra Bendixen

**Affiliations:** 1 Cognitive Systems Lab, Institute of Physics, Chemnitz University of Technology, Chemnitz, Germany; 2 Physics of Cognition Group, Institute of Physics, Chemnitz University of Technology, Chemnitz, Germany

**Keywords:** multistability, perception, auditory streaming, plaid-grating stimulus, pattern-component rivalry, eye-tracking, optokinetic nystagmus (OKN), support vector machine (SVM)

## Abstract

Multistable perception occurs in all sensory modalities, and there is ongoing theoretical debate about whether there are overarching mechanisms driving multistability across modalities. Here we study whether multistable percepts are coupled across vision and audition on a moment-by-moment basis. To assess perception simultaneously for both modalities without provoking a dual-task situation, we query auditory perception by direct report, while measuring visual perception indirectly via eye movements. A support-vector-machine (SVM)–based classifier allows us to decode visual perception from the eye-tracking data on a moment-by-moment basis. For each timepoint, we compare visual percept (SVM output) and auditory percept (report) and quantify the co-occurrence of integrated (one-object) or segregated (two-objects) interpretations in the two modalities. Our results show an above-chance coupling of auditory and visual perceptual interpretations. By titrating stimulus parameters toward an approximately symmetric distribution of integrated and segregated percepts for each modality and individual, we minimize the amount of coupling expected by chance. Because of the nature of our task, we can rule out that the coupling stems from postperceptual levels (i.e., decision or response interference). Our results thus indicate moment-by-moment perceptual coupling in the resolution of visual and auditory multistability, lending support to theories that postulate joint mechanisms for multistable perception across the senses.

## Introduction

Ambiguous stimuli can evoke bistable or multistable perception in all sensory modalities (Schwartz, Grimault, Hupé, Moore, & Pressnitzer, 2012). Visual examples—besides binocular rivalry (Wheatstone, 1838)—include dynamic moving plaids (Wallach, 1935) and static figures like the Necker Cube (Necker, 1832); the most prominent auditory examples are verbal transformations (Warren, 1961; Warren & Gregory, 1958) and auditory streaming (van Noorden, 1975). Under prolonged stimulation with such physically constant, yet ambiguous sensory information, two (bistability) or more (multistability) perceptual interpretations alternate in awareness – illustrating the on-going (re-)evaluation of sensory evidence (Leopold & Logothetis, 1999). The change from one dominating perceptual interpretation (percept) to another is referred to as a (perceptual) switch. Due to a variety of similarities in the reported switching patterns across modalities (Pressnitzer & Hupé, 2006), a universal principle or shared mechanism producing multistable perception across modalities has been suggested (Long & Toppino, 2004; Sterzer, Kleinschmidt, & Rees, 2009; Walker, 1978). Here we look at how similar multistable perception is across modalities from a new perspective: we study moment-by-moment coupling of perceptual interpretations across modalities by asking participants to directly report their perception of an ambiguous auditory stimulus, while their perception of an ambiguous visual stimulus is indirectly measured via their eye movements. This allows us to investigate perceptual bimodal moment-by-moment coupling without response interference.

Previous research linking several types of bi- or multistability within one modality or across modalities has often made use of inter-individual variation. There are huge inter-individual differences in certain properties of multistable perception (such as the rate of perceptual switching, the average duration of experiencing a percept, or the preference of one percept over another; Denham et al., 2014). A number of studies have asked whether these properties are consistent when exposing one individual to several different types of multistability. By means of correlation techniques, they looked at whether the relative position of an individual (i.e., being a fast or a slow switcher compared to a reference sample) reproduces across a range of multistable phenomena. The outcome of these studies has been somewhat mixed (Pressnitzer & Hupé, 2006; Kondo, Pressnitzer, Shimada, Kochiyama, & Kashino, 2018; Denham et al., 2018), depending among others on the type of stimuli that are being compared and on statistical power. Yet, more and more studies point towards the existence of such correlations (Brascamp, Becker, & Hambrick, 2018; Cao, Wang, Sun, Engel, & He, 2018; Einhäuser, da Silva, & Bendixen, 2020; Kondo et al., 2012; Pastukhov, Kastrup, Abs, & Carbon, 2019).

Interindividual correlations in perceptual bi- and multistability indicate that there are common factors affecting perceptual interpretations across stimuli and modalities. However, such common factors can be quite remote from the process of perceptual (re-)interpretation (Kleinschmidt, Sterzer, & Rees, 2012): Individual consistencies in multistable perception have been linked to personality traits (Farkas et al., 2016), neurotransmitter concentrations (Kashino & Kondo, 2012; Kondo, Farkas, Denham, Asai, & Winkler, 2017), and genetic factors (Schmack et al., 2013). The observed correlations might thus reflect more general perceptual or decision tendencies of a person; they are not necessarily indicative of a specific perceptual mechanism that governs the switching process on a moment-by-moment basis and that might or might not be shared across phenomena and modalities (Hupé, Joffo, & Pressnitzer, 2008).

Only few studies specifically address whether there is a common mechanism of perceptual switching on a moment-by-moment basis (Hupé et al., 2008). Such studies usually present two or more stimuli evoking perceptual multistability at the same time and ask participants to respond to both (or all) of them in parallel. Participants’ reports about their perception of each stimulus are then screened for an above-chance co-occurrence of switches or of corresponding percepts (Adams & Haire, 1959; Flügel, 1913; Grossmann & Dobbins, 2003; Hupé et al., 2008; Ramachandran & Anstis, 1983; Toppino & Long, 1987). A typical observation is that co-occurrences are observed, but their frequency is much lower than theoretically possible, which speaks against a strong coupling induced by a strictly common mechanism (Kubovy & Yu, 2012).

**Figure 1.**
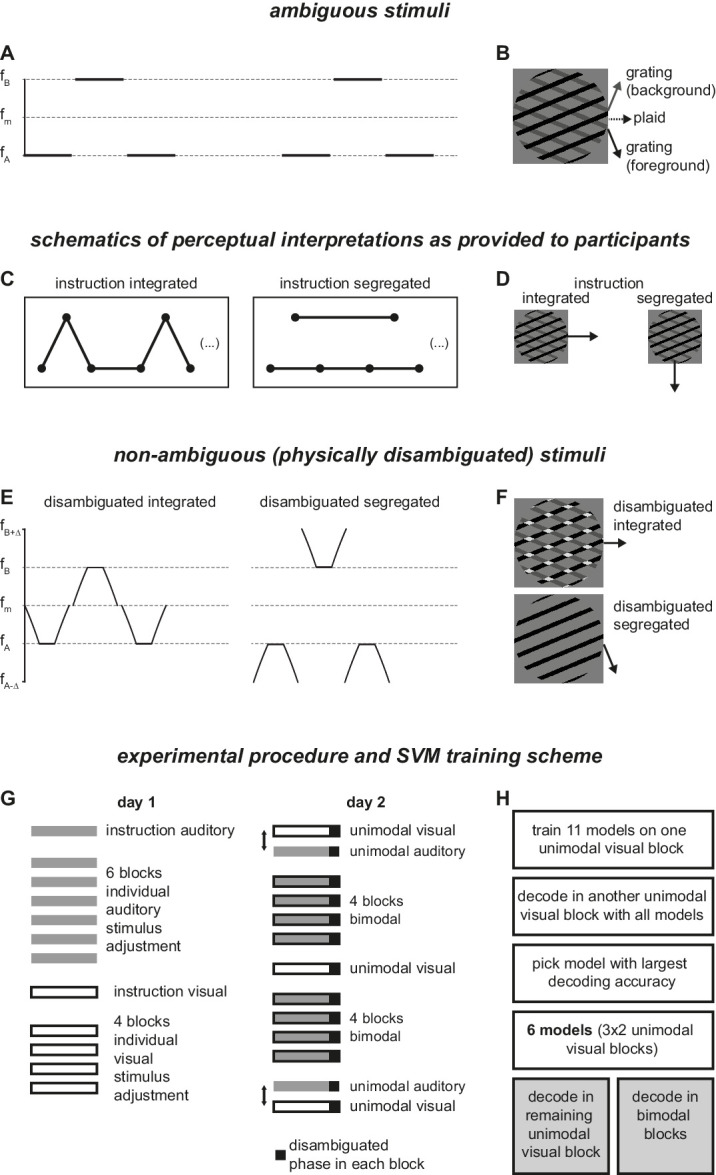
Stimuli and paradigm. (**A**) Auditory streaming stimulus. The frequency difference between tone A and B was adjusted individually between 1 and 14 semitones; f_m_ denotes their geometric mean (534 Hz in all participants). (**B**) Visual pattern-component rivalry stimulus. The opening angle was adjusted individually to a value of 35°, 45° (shown), or 55°. (**C**) Schematic of the two perceptual interpretations of the auditory streaming stimulus in A, as used for instructing participants. (**D**) Schematic of the two perceptual interpretations of the visual stimulus, as used for instructing participants. (**E**) Disambiguated auditory stimuli. Frequencies “f_A_,” “f_B_,” and “f_m_” match the individually adjusted tones in the multistable version (panel **A**), f_A__−__Δ_ and f_B+__Δ_ are chosen such that the distance in semitones (i.e., in log space) is identical, resulting in constant ratios in linear frequency space: f_A_/f_A__−__Δ_ = f_m_/f_A_ = f_B_/f_m_ = f_B+__Δ_/f_B_. (**F**) Disambiguated visual stimuli. (**G**) Order of blocks. Order of the unimodal blocks on day 2 is reversed in half of the participants. (**H**) Scheme of SVM training and testing; shaded boxes refer to reported results.

These prior studies all face the challenge of posing a dual-task requirement: Participants must monitor and report their perception for several stimuli at once. This involves interference at various processing levels, including nonperceptual stages such as decision and response interference. In the current study, we overcome this challenge by simultaneously presenting two stimuli evoking perceptual multistability (a visual and an auditory one) but asking for behavioral responses on only the auditory stimulus, while measuring perception of the visual stimulus via eye-tracking. We chose a visual stimulus whose perceptual alternatives are accompanied by markedly different eye movements, specifically the optokinetic nystagmus (OKN). The OKN is characterized by a sawtooth-like pattern of alternating slow and fast eye movements, where the slow phases follow the motion direction of the stimulus (Ilg, 1997). In multistability, the slow phases follow the currently *perceived* direction of motion; this has been well established for binocular rivalry (e.g., Fox, Todd, & Bettinger, 1975; Frässle, Sommer, Jansen, Naber, & Einhäuser, 2014; Naber, Frässle, & Einhäuser, 2011; Wei & Sun, 1998). In the present study, we used a visual pattern-component rivalry stimulus (Movshon, Adelson, Gizzi, & Newsome, 1985; Wallach, 1935; this specific version similar to Wilbertz, Ketkar, Guggenmos, & Sterzer, 2018), where two overlaid drifting gratings are presented that give rise to perceptual alternatives with distinctly different motion directions: either as one grating passing in front of the other (“component” or segregated percept) or as a unitary plaid (“pattern” or integrated percept) (Figure 1B, Supplementary Movie S1). Wilbertz and colleagues (2018) introduced a technique to enable moment-by-moment decoding from eye movements in response to the pattern-component rivalry stimulus using a support vector machine (SVM) classifier in a unimodal setting, such that the OKN gives an unobtrusive, en passant readout of the current percept. Here, we adapt this decoding method to our bimodal research question.

The simultaneously presented auditory stimulus was chosen according to a classical auditory streaming paradigm (van Noorden, 1975). This involves two tones (A and B) arranged in a repeating “ABA_” pattern, with “_” denoting a silent gap equivalent to tone B in duration (Figure 1A, Supplementary Movie S1). This arrangement can be perceived as coming from one sound source producing both the A and B tones (integrated percept, ABA_ABA_...) or from two sound sources producing the A and B tones in an interleaved manner (segregated percept, A_A_A_... and _B___B__...). Thus, in both modalities, there is an integrated (one visual plaid/one sound source) and a segregated (two visual components/two sound sources) interpretation. If the perceptual interpretation is coupled between audition and vision on a moment-by-moment basis, we should find co-occurrences of integrated and segregated percepts across modalities (Einhäuser, Thomassen, & Bendixen, 2020; Pressnitzer & Hupé, 2006).

When assessing moment-by-moment coupling between auditory and visual percepts, individual asymmetries in perceptual dominance for the stimulus in either modality (independent of the other) pose another important challenge. For example, if in one modality, percept A is dominant over percept B most of the time, and in the other modality percept C over D most of the time, there is a high co-occurrence probability for A and C, even in the absence of a true coupling. Vice versa, if the proportion of integrated/segregated percepts differs between the auditory and visual modality, this puts an upper limit to the times in which co-occurring percepts can be observed—even under ideal coupling, perfect (100%) correspondence cannot be reached. To avoid extreme asymmetries and thus increase statistical power for finding moment-by-moment coupling if there is any, we pre-adjusted the stimulus parameters to draw near a 50/50% distribution of integrated and segregated percepts, separately for each individual and in each modality. The adjustments were based on changing parameters known to affect the relative proportion of integrated and segregated percepts: the frequency difference between the A and B tones (Denham, Gyimesi, Stefanics, & Winkler, 2013; van Noorden, 1975), and the enclosed angle (“opening angle”) between the gratings (Carter, Snyder, Fung, & Rubin, 2014; Hedges, Stocker, & Simoncelli, 2011; Hupé & Pressnitzer, 2012; Wegner, Grenzebach, Bendixen, & Einhäuser, 2021). Any remaining asymmetries were accounted for in the analysis.

With this stimulus configuration, which is tailored toward a fair test of bimodal moment-by-moment coupling in perceptual multistability, we investigate the hypothesis that integrated percepts and segregated percepts in the two modalities tend to co-occur. This, in turn, would indicate a partly common, supra-modal mechanism in control of perceptual multistability.

## Methods

### Participants

A total of 25 volunteers (nine men, 16 women; age 20–31; mean age 23.4 years) were recruited from the Chemnitz University of Technology community and participated in the experiment. After applying the a priori defined inclusion criteria (see below: at least 70% hit rate for catch trials in the unimodal blocks and at least 70% decoding accuracy of the visual percept from the eye-tracking data in the unimodal visual blocks), data of 16 participants (five men, 11 women; age 20–31; mean age 23.6 years) were included in the main analysis.

#### Number of participants and inclusion and stopping criteria

The number of participants to be included in the analysis had been decided to be 16 before the experiment. Directly after the second session of each individual, we determined hit rates in the unimodal blocks and decoding accuracy in the unimodal visual blocks while remaining blind to all other data (except for a visual inspection of the eye-tracking data quality). If a participant failed to meet the 70% criterion for any of the measures, a replacement was added until 16 participants met all inclusion criteria. In the analysis, all criteria are first considered separately for all participants, and data for all participants are reported for each criterion.

### Setup

Experiments were conducted in a sound-attenuated room with no light source other than the visual display. All visual and auditory stimuli were presented using a 22.5-inch “ViewPixx Full” LCD display (VPixx Technologies Inc., Saint-Bruno, QC Canada), which was located 55 cm from the participants’ head. The monitor operated at 120 Hz and 1920 × 1080 pixels for the visual display and 48 kHz for its auditory output. The auditory stimuli were presented through “HD25-1-II, 70 Ohm” headphones (Sennheiser, Germany). Responses were recorded by the display device using the manufacturer's “ResponsePixx” 5-button box, whose buttons are arranged in a “plus”-shaped pattern (one in the middle, one to each direction). Gaze direction of the right eye was recorded at 1000 Hz by an “EyeLink-1000 Plus” eye-tracking system (SR Research, Ottawa, ON, Canada) in “tower mount” configuration. The eye tracker was calibrated and the calibration validated before each visual block on day 1 and before each block on day 2. Participants rested their head on a padded chin rest and a padded forehead rest to minimize head movements during the experiment. For control of the experiment, Matlab (The MathWorks, 2019) and its Psychophysics toolbox (Brainard, 1997; Pelli, 1997) and Eyelink toolbox (Cornelissen, Peters, & Palmer, 2002) were used.

### Stimuli and tasks

In the main part of the experiment, participants were simultaneously presented with an auditory streaming stimulus (Figure 1A) and a visual pattern-component rivalry stimulus (Figure 1B, Supplementary Movie S1). During this *bimodal* condition, participants reported their auditory perception as either integrated or segregated (Figure 1C) while merely looking at the visual stimulus. Their visual perception was decoded from their eye movements with an SVM classifier (Wilbertz et al., 2018). To train the SVM classifier and to evaluate its ability to decode the visual percept, participants additionally conducted a *unimodal visual* condition, where only the visual stimulus was presented and participants continuously reported their visual percept as integrated (plaid/pattern) or segregated (gratings/components) (Figure 1D). To have a comparison for assessing the effects of bimodal presentation, participants further conducted a *unimodal auditory* condition. Analysis was based exclusively on these bimodal and unimodal conditions recorded in one session (“day 2” in Figure 1G).

Unimodal conditions were also used in a separate session (“day 1” in Figure 1G) conducted on a different day prior to the main session. The purpose of the first session was to familiarize participants with the stimuli and to ensure a roughly equal amount of segregated and integrated multistable percepts in both modalities by individually adjusting the parameters of the visual and auditory stimuli for each participant (for details see section *Individual Adjustment of Stimuli*).

In all conditions (unimodal and bimodal) of day 2—but not in the individual adjustment session (day 1)—the multistable presentation lasted 180 seconds (“multistable part”) and was seamlessly followed by a “disambiguated part” of about 30 seconds, which was used for response verification akin to catch trials (Figure 1G). In this part, participants continued to report as in the multistable part (i.e., their visual perception in the unimodal visual condition, their auditory perception in all other conditions), but either one of the stimuli was disambiguated. This pertained to the visual stimulus (Figure 1F, Supplementary Movie S2) in the unimodal visual condition and half of the bimodal-condition blocks, and to the auditory stimulus (Figure 1E, Supplementary Soundfile S3) in the unimodal auditory condition and the other half of the bimodal-condition blocks. The disambiguated stimulus alternated between suggesting an integrated percept and suggesting a segregated percept, such that in each disambiguated part each percept was suggested twice (for about 7.5 seconds).

#### Auditory stimuli

As ambiguous stimulus, we used a variant of the auditory ABA_ streaming stimulus (van Noorden, 1975). Pure tones of two different frequencies (f_A_ and f_B_) were presented in alternation for 130 ms each, followed by a 20-ms silence (i.e., at a stimulus onset asynchrony of 150 ms), where every other “B” tone was replaced by silence (Figure 1A). Tones had an A-weighted sound-pressure level of 70 dB(A), as measured by a hand-held “Type 2270” sound level meter and “Type 4101-A” binaural microphones (Bruel & Kjaer, Denmark). Tones started and ended with 5-ms raised-cosine amplitude ramps. Frequencies were adjusted per individual but the center frequency - the geometric mean f_m_ between f_A_ and f_B_ - was fixed at 534 Hz. That is, they had equal ratios on a linear scale (f_m_/f_A_ = f_B/_f_m,_), corresponding to equal distances on a log scale (log f_m_ − log f_A_ = log f_B_ − log f_m_).

As catch trials to check the validity of participants’ reports, we constructed disambiguated versions of the auditory stimulus (as in Grenzebach, Wegner, Einhäuser, & Bendixen, 2021). To simulate an integrated percept, the 130-ms tones were replaced as follows (Figure 1E, left; Supplementary Soundfile S3): tone A was replaced by a chirp that started at the center frequency f_M_, was reduced linearly to f_A_ over 30 ms, remained at f_A_ for 70 ms and then rose again to f_M_ over 30 ms; tone B was replaced by the reverse pattern (30 ms rise from f_M_ to f_B_, 70 ms f_B_, 30 ms drop to f_M_). To simulate a segregated percept, the reverse pattern was applied (Figure 1E, right; Supplementary Soundfile S3): tone A was raised from a frequency f_A−Δ_ to f_A_ and dropped back to f_A−Δ_, whereas tone B started at f_B+Δ_, dropped to f_B_ and then rose again to f_B+Δ_. In both cases, f_A_, f_M_, and f_B_ matched the ambiguous case, f_A−Δ_ and f_B+Δ_ were chosen such that the distance in log frequency (semitones) between all subsequent frequency levels were identical, that is:

$$\begin{array}{ccc} \;\log\;f_{A}-\log\;f_{A-\Delta }\; & = & \log\;f_{m}-\log\;f_{A}=\log\;f_{B}-\log\;f_{m}\\ & = & \log\;f_{B+\Delta }-\log\;f_{B} \end{array}$$

which is equivalent to

$$\begin{array}{c} f_{A}/f_{A-\Delta }=f_{m}/f_{A}=f_{B}/f_{m}=f_{B+\Delta }/f_{B} \end{array}$$

in linear space.

#### Visual stimuli

As visual stimulus evoking multistability, we used two overlapping drifting square-wave gratings (Figure 1B, Supplementary Movie S1) akin to the one used in Wilbertz et al. (2018), except that the drift direction was rotated by 180° and that the angle between the gratings (“opening angle”) was adjusted per individual. The orientation of the pattern was such that the plaid formed by the gratings drifted always horizontally to the right; that is, both gratings formed the same angle with the horizontal but with opposite signs. The gratings were black (<0.1 cd/m^2^) and dark gray (6 cd/m^2^) on a light gray (12 cd/m^2^) background and presented in a circular aperture with a diameter of 7.5 degrees of visual angle. Gratings had a duty cycle of 0.28 and a spatial frequency of 0.8 cyc/deg and drifted at 1.9 deg/second perpendicular to their orientation, the black grating downward and the gray grating upward. Intersections between the gratings were colored in black, such that the black grating is typically perceived to drift on top of the gray grating (i.e., in the foreground). Disambiguated visual stimuli (Figure 1F, Supplementary Movie S2) were formed by either coloring the intersections white (24 cd/m^2^) to induce an integrated (plaid) percept, or by removing the gray grating to induce a segregated (component) percept. During unimodal auditory blocks, a dark gray disc was presented in place of the aperture on a light gray background.

### Experimental procedure

#### Day 1—instruction and paradigm

The experiment was split over two sessions that were conducted on separate days with on average 2.5 days in between (Figure 1G). The first session started with a demonstration of extreme cases of the auditory stimulus to demonstrate the possible percepts, using a frequency difference of one semitone to demonstrate the integrated percept, of 13 semitones to demonstrate the segregated percept, and seven semitones to demonstrate multistability. Afterward participants were instructed about their response mapping. Of the included 16 participants, in nine the left button mapped to the segregated percept and the middle button to the integrated percept, for the remaining seven the mapping was reversed. Participants then practiced the response mapping with the extreme examples. Once they and the experimenter were confident that the percepts and the mapping were understood, they proceeded to the individual adjustment of the auditory stimulus. After this adjustment, the visual stimuli were introduced, and the response mapping—bottom button for the segregated (component) percept moving downward, right button for the integrated (plaid) percept moving rightward—was practiced with the multistable and the two disambiguated examples, each at 45° opening angle. As the relative location of the button corresponded to the perceived direction of motion, the response mapping was kept identical for all participants to avoid any form of compatibility effects. After instruction and familiarization with the response mapping, the individual adjustment of the visual stimuli commenced.

#### Day 1—individual adjustments of stimuli

Separately for the visual and the auditory stimulus, we intended to achieve a balance between integrated and segregated percepts, that is, to prevent strong asymmetries towards either percept. Since the tendency towards integration or segregation shows large inter-individual variability (Denham et al., 2014), this adjustment has to be conducted on an individual level. To this end, we presented different versions of the stimuli evoking multistability in two-minute blocks. For the auditory stimulus, we started at a frequency difference of seven semitones. If there was an abundance of integrated percepts, the frequency difference was increased in the subsequent block, if there was an abundance of segregated percepts, the frequency difference was decreased. The degree of increase or decrease depended on the strength of the observed asymmetry. This was repeated for four blocks, then a final setting was decided upon and one more block with this frequency distance was run. This frequency distance was then also used throughout day 2. For the 16 included participants, these frequency distances ranged between two and 10 semitones. Similarly, we adjusted the visual stimulus. Here, we presented the 45° opening angle stimulus for two 2-minute blocks and the 35° and the 55° for one 2-minute block each. The opening angle with the best balance between integration and segregation was then picked and used throughout day 2.

#### Day 2—main experiment

The main session was run on day 2 and consisted of eight bimodal blocks, three unimodal visual blocks, and two unimodal auditory blocks (each with a 180-second multistable part and a 30-second disambiguated part). The unimodal visual blocks were presented at three distinct timepoints: before, halfway through, and after the bimodal condition. The unimodal auditory blocks either preceded or followed the first and the last unimodal visual block. In half of the participants, the order was visual-unimodal, auditory-unimodal, four blocks bimodal, visual-unimodal, four blocks bimodal, auditory-unimodal, visual-unimodal, in the other half the order of visual-unimodal and auditory-unimodal at beginning and end was reversed (Figure 1G).

#### Ethics statement

All experimental procedures conformed to the principles laid out in the Declaration of Helsinki and were evaluated by the applicable ethics board (Ethikkommission der Fakultät für Human- und Sozialwissenschaften) at Chemnitz University of Technology, who waived an in-depth evaluation (case no. V-219-15-AB-Modalität-14082017).

### Data analysis

All analyses are based on the data recorded in the main session (day 2). All data and analysis code are available at https://doi.org/10.17605/OSF.IO/SRQ5Y.

#### Behavioral data

For the disambiguated part of each block, where—unlike for the multistable part—correctness of the response can meaningfully be defined, participants’ behavioral responses were scored as correct or incorrect at each timepoint. To be included in the main analyses, a participant had to report the correct percept for at least 70% of the time they reported one percept exclusively in the disambiguated sequences (“hit rate”). The 70% criterion had to be passed separately for the visual and auditory conditions. Only the unimodal blocks were used for defining the exclusion criterion. Setting this as a baseline allowed us to check whether participants’ ability to report faithfully changes during bimodal blocks.

For the multistable part of each block, the summed time during which one of the two percepts (integrated, segregated) was exclusively reported was collected, and its percentage relative to the overall stimulus time was calculated. The proportions of integrated and segregated percepts were then calculated relative to the summed time of exclusive reporting of one perceptual state (i.e., they added up to 100%, making one of them a redundant measure).

#### Eye movement data

Our procedures for the extraction of features and for training the SVM classifier closely followed the approach proposed in Wilbertz et al. (2018). The key underlying idea is that when observing a pattern-component rivalry stimulus, the slow phase of the OKN aligns with the perceived motion. As the horizontal motion component for a horizontally moving plaid (pattern) is larger than for a grating (component) that drifts mostly vertically, the velocity of the slow phases and to some extent their duration, depend on the percept. Because we were not limited by the constraints of a functional-magnetic-resonance-imaging-compatible eye-tracking setup, we decided to sample the features at higher resolution than Wilbertz et al. (2018). Because we included three independent unimodal-visual blocks into our design, we also could replace their leave-one-out (10-fold cross-validation) procedure and instead use independent blocks as sets for training, for parameter optimization, and for evaluation.

#### SVM classifier and feature extraction from eye-movement data

**Figure 2.**
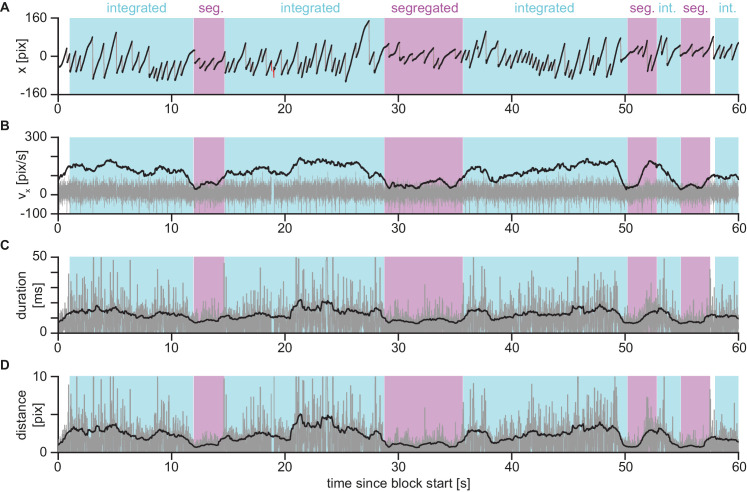
Eye-movement variables to compute SVM features. (**A**) First minute of horizontal eye position during a representative unimodal visual block. *Black*: slow phases, *gray*: fast phases (as determined by saccade detection algorithm), *red*: blink detection around missing data. (**B**) *Gray*: velocity computed as difference between subsequent datapoints (scaled down by a factor of 10 relative to the scale on y-axis) after removing fast phases and blinks from trace in panel A; *black*: sliding average of velocity data using a 1-s time window. (**C**) *Gray*: number of successive samples in direction of fast phase; *black*: sliding average over gray data. (**D**) *Gray*: distance covered by successive samples in direction of OKN fast phase, *black*: sliding average over gray data. In all panels, periods in which the participant reported an integrated percept are marked in cyan, segregated reports in magenta and periods when no or both buttons were pressed are left unmarked. See Figure 5 in the Appendix for representative sections of the time series zoomed-in.

As did Wilbertz et al. (2018), we extracted the time courses of three variables from the raw horizontal eye-position data (Figure 2). We separated fast phases and slow phases of the OKN (Figure 2A) and extracted the same features as Wilbertz et al.—a smoothed slow-phase velocity (Figure 2B), a robust measure of the duration of OKN fast phases (Figure 2C) and of the distance covered by the fast phases (Figure 2D). Shifting these measures in 100-ms steps from −2.0 to 0 seconds relative to the analyzed timepoint resulted in a 63-dimensional feature vector at each timepoint (see Appendix for details).

Using the 63-dimensional feature vector at 100 Hz sampling, we trained an SVM classifier with the libSVM implementation for Matlab (https://github.com/cjlin1/libsvm) in version 3.23 (Chang & Lin, 2011). We used a linear kernel and the same 11 cost parameter settings (0.0001, 0.0005, 0.001, 0.005, 0.01, 0.1, 1, 2, 5, 10, 100) as in Wilbertz et al. (2018). Of the three unimodal visual blocks each participant had performed, one block was used to train the SVM classifier with different cost-parameter settings (11 models per block and participant), another block to choose the optimal cost parameter (i.e., retaining the model that showed highest decoding accuracy for this block), and the third block to evaluate the performance of the model with the thus chosen cost parameter (in terms of decoding accuracy, see below). By this setting, the training set, the set for choosing the cost parameter and the set used for evaluation are independent. Because there was no a priori reason to assign one of the three roles (training, parameter optimization, evaluation) to a specific block, the role of the three unimodal visual blocks was permuted within a participant, such that six different models per participant were retained for further use^1^ (Figure 1H).

#### Decoding accuracy

Throughout this study, the term *decoding accuracy* refers to a class-adjusted accuracy which first computes the accuracy for both classes (i.e., percepts) separately:

$$\begin{array}{ccc} & & accuracy_{segregated}\\ & & \;=\frac{\#timepoints\;model\;predicts\;segregated\;and\;segregated\;is\;reported}{\#timepoints\;segregated\;is\;reported} \end{array}$$

$$\begin{array}{ccc} & & accuracy_{integrated}\\ & & \;=\frac{\#timepoints\;model\;predicts\;integrated\;and\;integrated\;is\;reported}{\#timepoints\;integrated\;is\;reported} \end{array}$$

where “#” denotes “number of.” The accuracy for the two percepts is then averaged, making the measure insensitive to asymmetric distributions in ground truth (i.e., the reported percepts):

$$\begin{array}{c} accuracy=\frac{accuracy_{segregated}+accuracy_{integrated}}{2} \end{array}$$

This class-adjusted decoding accuracy (also called balanced decoding accuracy) is insensitive to asymmetries in the reported percepts. This ensures that chance level (the expected accuracy if the model's classifications were based on random guessing) is always at 50%; note, however, that the theoretical and expected upper and lower bounds are not symmetric around this chance level (see Supplementary Text).

Decoding accuracy was computed in the unimodal visual blocks to reflect how well the SVM classifier decoded the visual percept from the eye-tracking data. The timepoints of reported percepts in the above formula thus refer to reports of *visual* segregation or integration for this analysis. To be included in the main analyses, a participant had to reach a decoding accuracy of 70% in the unimodal visual blocks.

An analogous definition of decoding accuracy was applied in the bimodal blocks to reflect the consistency between the decoded visual and the reported auditory percept: While still the visual stimulus is decoded, the labels (i.e., segregated or integrated percept reported) stem from the auditory report. The timepoints of reported percepts in the above formula thus refer to reports of *auditory* segregation or integration for this analysis. The thus defined consistency between reported auditory and decoded visual percept was computed separately for each of the six models and eight bimodal blocks; these 48 values were then averaged within a participant.

#### Theoretical and expected bounds on consistency

The expected maximum for the consistency between reported auditory and decoded visual percept in bimodal blocks is limited by several factors: by the accuracy of the visual decoding, by the ability to faithfully report one's auditory percept, and by any remaining asymmetries in the distribution of the two classes (percepts) across modalities. Perceptual asymmetries lead to both upper and lower bounds of consistency: if one of the percepts is more frequent in one modality than the other, the overall consistency cannot reach 100%; in turn, it cannot reach 0%, either. The observed values (proportion of integrated/segregated within each modality) give a theoretical maximum of the true underlying perceptual consistency that an individual can reach. Independently of these boundaries, the visual decoding accuracy and auditory reporting accuracy pose additional limitations in terms of the consistency that can be expected to be empirically observed. Specifically, when the true auditory percept perfectly matches the true visual percept, the visual decoding will be consistent to the auditory report if either both are correct or both are incorrect (as there are only two classes, i.e., percepts). For each individual, visual decoding accuracy was estimated from their decoding accuracy in the unimodal visual blocks, and auditory reporting accuracy was estimated from their hit rate in the auditory catch trials of the bimodal blocks. Under the reasonable assumption that at any given timepoint, the ability of the SVM to decode correctly is independent of the ability of the participant to report their percept correctly, bounds for the maximum and minimum consistency can be calculated, both in terms of theoretical limits (constrained by the perceptual asymmetry) and in terms of expected limits (in addition constrained by reporting and decoding accuracy). Analytical expressions for these bounds as a function of decoding accuracy, reporting accuracy and percept asymmetry are derived in the Supplementary Material. Note that chance level for the class-adjusted accuracy remains at 50% for any combination of these values. We contrast our empirically observed consistency values statistically against this 50% reference and compare them descriptively against the upper and lower bounds.

### Statistical analysis

To test whether a population mean is significantly different from chance, we employed one-sample *t*-tests. To compare two conditions across observers, we used *t*-tests for dependent samples (paired *t*-tests). In all cases, two-tailed tests at an alpha level of 5% were used.

## Results

### Unimodal blocks: Disambiguated part

The 180-second multistable part of each main block was followed by a 30-second disambiguated part. For stimuli evoking multistability, by definition, the reported percept is subjective and there is no “correct” or “incorrect” response. For the disambiguated parts, in contrast, correctness is meaningful and responses can be validated whenever the participant reports on the disambiguated modality. The disambiguated parts in unimodal visual blocks and unimodal auditory blocks thus served for response verification. To be included in the main analyses, a participant had to show a hit rate of at least 70% for both visual and auditory catch trial sequences. Of the 25 participants, all fulfilled this criterion for the visual unimodal blocks (mean hit rate: 91.5%, standard deviation, *SD* = 2.6%, range 85.4%–95.4%). In the auditory unimodal blocks, seven participants failed to reach this criterion, while the remaining 18 were above the 70% threshold (mean hit rate for N = 25: 76.4%, *SD* = 14.0%, range 46.4%–89.8%; mean hit rate after exclusion, i.e., for N = 18: 84.0%, *SD* = 5.2%).

### Unimodal visual blocks: Decoding accuracy

**Figure 3.**
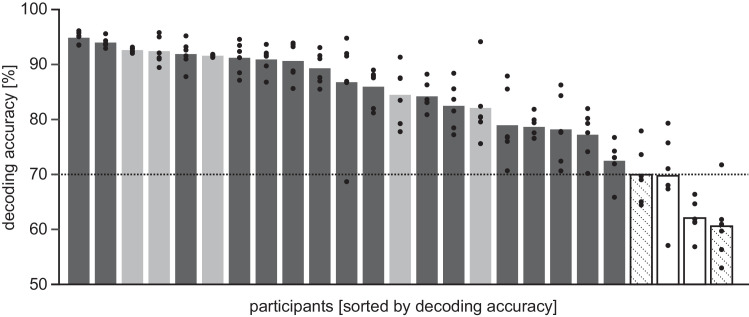
Individual decoding accuracy in unimodal visual blocks and exclusion criteria. Bars denote the mean over the six decoding models, black circles the individual models. Data are sorted by decoding accuracy; horizontal lines indicate chance level (50%) and a priori exclusion criterion (70%). *Dark* *gray* *bars*: included participants, *open bars*: participants excluded based on decoding accuracy, *light* *gray* *bars*: individuals excluded based on hit rate for auditory catch trials; *hatched bars*: exclusion based on both criteria.

For each individual, we used each of the six SVM classifiers to decode the percept in the unimodal visual block the specific classifier had neither been trained nor optimized on. When averaging over the six models per individual, mean class-adjusted decoding accuracy across all participants was 82.9% (*SD*: 9.9%) and thus well above chance [*t*(24) = 16.67, *p* < 0.001; one-sample *t*-test against 50%, Figure 3]. This conceptually replicates the finding that this SVM method can be applied to robustly assess the percept of a pattern-component rivalry stimulus on a moment-by-moment basis (Wilbertz et al., 2018).

Although all participants showed decoding accuracies above chance (Figure 3), four participants remained below the a priori defined inclusion criterion of at least 70% in this accuracy measure and were excluded from further analysis. Two of these had not met the 70% criterion on hit rates in auditory catch trials either. Hence, 16 participants were retained for further analysis.

### Unimodal blocks: Multistable part

In the unimodal visual blocks, participants reported one of the two percepts exclusively for 97.2% (*SD* = 2.7%) of the multistable part. On average the proportion of segregated percepts was about half (48.2%, *SD* = 6.4%) and not significantly different from 50%, *t*(15) = 1.10, *p* = 0.29, with the individuals ranging from 35.6% segregated to 61.7% segregated (i.e., 38.3% to 64.6% integrated) reports. In the unimodal auditory blocks, participants reported one of the two percepts exclusively for 97.1% (*SD* = 3.0%) of the multistable part. On average the proportion of segregated percepts was 59.7% (*SD* = 18.3%) and not significantly different from 50%, *t*(15) = 2.12, *p *= 0.051, with the individuals ranging from 32.8% segregated to 90.0% segregated reports. Together, this shows that the adjustment procedure to yield about equal numbers of segregated and integrated percepts was successful in both modalities for the group average (though in the auditory modality, there was a trend toward more segregation). However, on the individual level, substantial deviations from the targeted 50/50% distribution were observed in both modalities. Importantly though, the individual adjustment successfully abolished any correlations between the proportions of integrated/segregated percepts in the visual and auditory modalities across participants (correlation of unimodal-visual and unimodal-auditory proportions: *r* = 0.05, *p* = 0.851, N = 16; correlation of unimodal-visual and bimodal-auditory proportions: *r* = 0.08, *p* = 0.770, N = 16).

### Bimodal blocks: Behavioral data

In all bimodal blocks, participants reported their auditory percept throughout. In half of the blocks, the disambiguation in the last 30 seconds pertained to the auditory stimulus. Hence, these four parts can serve as additional catch trials for response verification. Hit rate was 87.1% (*SD* = 3.2%) on average, which was slightly larger than the hit rate in the unimodal auditory condition for the 16 included participants (mean, *M* = 84.1%, *SD* = 5.4%, *t*(15) = 2.38, *p* = 0.031, paired *t*-test). This indicates that the presence of the visual stimulus does not disrupt the ability to report the auditory percept; if anything, there is a slight improvement.

In the multistable parts, the proportion of segregated reports in the bimodal blocks (59.6%, *SD* = 15.8%) did not differ from that in the unimodal auditory blocks (59.7%, *SD* = 18.3%; *t*(15) = 0.06, *p* = 0.96). Likewise, the median duration of each perceptual phase in the bimodal blocks (mean across participants: 8.36 seconds, *SD* = 6.37 seconds) did not differ from that in the unimodal auditory blocks (*M* = 7.99 seconds, *SD* = 5.38 seconds; *t*(15) = 0.35, *p* = 0.73). This indicates that the presence of a visual stimulus does not alter auditory multistability *on average* but leaves open whether there is a moment-by-moment influence of one modality on the other.

### Bimodal blocks: Relating the decoded visual percept to the auditory report

**Figure 4.**
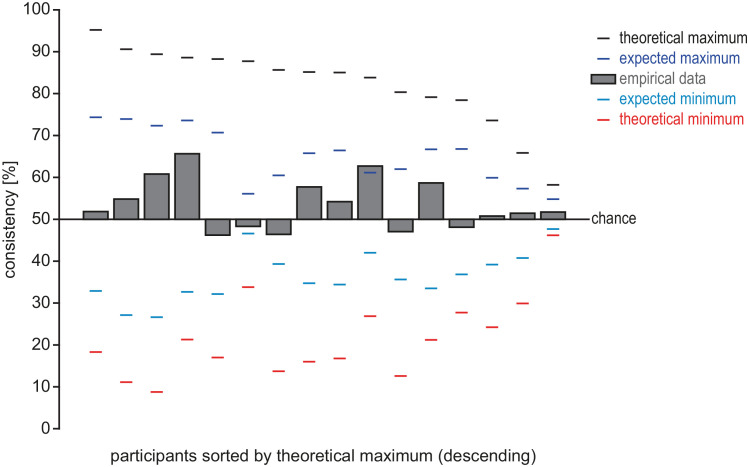
Consistency of auditory and visual percepts. *Bars*: consistency between the reported auditory percept and the decoded visual percept, as measured by the “decoding” of the auditory percept using the visual model. Individuals are sorted by their theoretical maximum consistency. *Black line*: theoretical maximum consistency based on asymmetries between visual and auditory stimulus percept distributions; *blue line:* expected maximum consistency based on the combination of percept asymmetries with decoding and reporting accuracy; *red and cyan line:* theoretical and expected minimum consistencies based on asymmetries and accuracies.

In the bimodal blocks, participants reported their auditory perception while their visual perception was decoded using the SVM classifier trained on the unimodal visual blocks. To arrive at a measure of moment-by-moment consistency between visual and auditory perception, we used the SVM classifier to “decode” the auditory percept. That is, we used the auditory report as labels for testing the SVM, as if they were referring to the corresponding visual percept—visual integration (plaid) to auditory stream integration, visual segregation (gratings) to auditory stream segregation. If the auditory report and the visual percept were perfectly identical (whenever a participant reports auditory integration they perceive a plaid, whenever a participant reports auditory segregation they perceive a grating), this measure would be equal to the decoding accuracy in the unimodal visual blocks (Figure 3). Conversely, if the visual and the auditory percept were entirely unrelated on a moment-by-moment basis, this measure would be at chance (50%; here it is especially critical that the accuracies are first determined by class [percept] and then averaged, as otherwise the chance-level would be inflated if an individual has common asymmetries in both modalities). We computed the consistency between reported auditory and decoded visual percept for each of the six models and eight bimodal blocks and then averaged these 48 values within a participant. The resulting consistency across observers (Figure 4; mean: 53.5%, *SD* = 6.1%, range 46.2% to 65.7%) was significantly above chance, *t*(15) = 2.33, *p* = 0.034 (one-sample, two-tailed *t*-test against 50%). This demonstrates moment-by-moment coupling between visual and auditory multistability.

Although the consistency between decoded visual and reported auditory percept is significantly above chance, its absolute value may seem comparably small. It should be noted, however, that the expected maximum for this value is considerably below 100%. Separately for each participant, we computed the *theoretical* maximum consistency based on their asymmetry of auditory and visual percepts, as well as an *expected* maximum consistency additionally taking their visual decoding accuracy and auditory reporting accuracy into account (Figure 4, black for theoretical maximum value, blue for expected maximum value; for derivation, see Supplementary Text). We also computed the converse bounds, that is, a theoretical minimum consistency and expected minimum consistency under the assumption that the percepts are as inconsistent as possible under the given constraints (Figure 4, red for theoretical minimum value, cyan for expected minimum value; for derivation, see Supplementary Text). Values below chance (50%) approaching the minimum consistency would indicate systematic “anti-coupling” of percepts in the two modalities. When compared to their individual expected bounds, many participants’ consistency is not only above chance (50%), but also covers a sizable fraction of the range between chance and their expected maximum. Relative to the possible range given by their perceptual asymmetry, decoding and reporting accuracy, some participants show substantial bimodal consistency even on an individual level; in contrast, no individual shows a strong indication of anti-coupling (i.e., values substantially below chance level).

## Discussion

We studied moment-by-moment correspondence of perceptual interpretations across modalities by having participants directly report their perception of an ambiguous auditory stimulus (auditory streaming; van Noorden, 1975), while measuring their perception of an ambiguous visual stimulus (pattern-component rivalry) indirectly via their eye movements (Frässle et al., 2014; Wilbertz et al., 2018). In line with the hypothesis of a shared mechanism contributing to multistable perception across modalities, our results demonstrate moment-by-moment coupling between visual and auditory multistability.

The most important advancement relative to previous studies on moment-by-moment coupling in multistability (Adams & Haire, 1959; Flügel, 1913; Grossmann & Dobbins, 2003; Hupé et al., 2008; Ramachandran & Anstis, 1983; Toppino & Long, 1987) is the elimination of response interference. This was possible in the current study by measuring only one of the percepts behaviorally. With behavioral measurements of two or more percepts in parallel, it remains unclear whether the observed coupling is an effect of perception per se, or rather related to *monitoring* the percepts as per task instruction. This distinction is critical, as distinct neural substrates subserve either function (Frässle et al., 2014). Here, in the main (bimodal) condition, only one modality (audition) was task-relevant and reported, while the percept in the other modality (vision) was inferred from eye movements.

Besides this distinction at the level of mechanisms (perception per se versus monitoring of perception), the indirect measurement of the visual percept is also advantageous from the perspective of task design. Giving behavioral reports for two or more stimuli in parallel is challenging for the participants and might undermine the reliability of their reports (Conrad, Vitello, & Noppeney, 2012). In addition to the challenge of a dual task, response mapping must be carefully chosen to avoid compatibility effects at the level of the response effector or response action (button press vs. release). Our main behavioral measurements involved reporting the auditory percept only. In additional unimodal visual blocks, we had participants report their visual percept (for choosing suitable stimulus parameters and for training the SVM). These visual blocks constituted a small fraction (three out of 13 blocks in the main part, i.e., on the second day), and the response buttons were different from those for reporting the auditory percepts. Moreover, the relative mapping of the buttons to integrated and segregated auditory percepts were counterbalanced across participants to avoid any systematic interference effects between visual and auditory response mapping.

One might argue that taking behavioral reports for different (auditory/visual) stimuli in separate blocks, with the main (bimodal) condition involving the simultaneous presentation of auditory and visual stimuli, might be confusing for participants and thus not necessarily constitute an advantage over dual-task requirements (Hupé et al., 2008). Indeed, if participants had erroneously responded to the visual rather than the auditory stimulus during bimodal blocks, this might produce an apparent bimodal coupling of percepts which would in fact represent a misunderstanding of task requirements. To make sure that participants responded to the auditory (not the visual) stimulus during bimodal blocks, we appended catch trial segments with disambiguated versions of either the auditory or the visual stimulus. Unlike the hit rate for the auditory disambiguated phases (87.1% on average for the included N = 16), the “pseudo” hit rate for the visual disambiguated phases (i.e., the hit rate under the assumption that participants wrongfully reported their visual percept) was at 51.1% indistinguishable from chance, *t*(15) = 0.73, *p* = 0.48. This indicates that perceptual reports were made in response to the auditory stimulus—that is, participants complied with the instruction.

Having one modality as task-irrelevant (which is possible through the visual no-report measure; Wilbertz et al., 2018) also prevents participants from applying certain response strategies that are difficult to avoid in the dual-task case. Such response strategies might artificially increase (e.g., deliberate switching at the same time) or decrease (e.g., deliberate abstention from switching at the same time) the probability to find moment-by-moment coupling. Eliminating response strategies and response criteria (i.e., when to transform the constantly fluctuating perceptual information into a change of the binary integrated/segregated response) is notoriously difficult (Brascamp et al., 2018). In the current study, using the OKN to assess visual perception allows for a criterion-free measurement, because the OKN is a reflex under little to no volitional control. Response criteria in the auditory reports remain, but they can no longer increase the measure of bimodal coupling—thus if anything, they act against the hypothesis.

The advantages of this indirect measure of visual perception come at the cost of a possibly non-perfect moment-by-moment readout. We adopted a recently proposed procedure using SVM (Wilbertz et al., 2018) for classifying the visual percept based on eye movements. The mean accuracy across all participants was 82.9%, which is of the same order as the 87.66% value reported by Wilbertz et al. (2018) and thus conceptually replicates their work. The classification is thus sufficiently robust for the current analyses. The main feature that allows this robust decoding is the design of the specific pattern-component rivalry stimulus (Wilbertz et al., 2018), which is constructed in a way that the segregated (component) percept comes with a clear separation into foreground and background grating, and thus with a unanimous motion direction. Relative to other versions of pattern-component rivalry (Einhäuser et al., 2020; Wegner et al., 2021), where the segregated percept consists of two equally strong components and thus two simultaneously perceived motion directions, this gives a much better OKN distinction between integrated (plaid/pattern) and segregated (component) percepts. Together with the SVM-based classifier (Wilbertz et al., 2018), this led to a robustness of moment-by-moment readout that allowed us to go substantially beyond our own previous investigations: In Einhäuser et al. (2020), we had likewise taken direct measures of auditory perception and OKN-based indirect measures of visual perception. We showed that the average OKN gain in the motion direction of the integrated visual percept was higher during integrated than during segregated auditory percepts. We were, however, not able to show that this translates into coupling on a moment-by-moment level, and we were not able to show any coupling for the converse case (i.e., in the motion direction of the segregated visual percept, because there were two motion directions as denoted above). The moment-by-moment coupling observed in the current study thus considerably strengthens the evidence in favor of a partly supra-modal mechanism of perceptual multistability.

Another important difference between the current study and Einhäuser et al. (2020) is the individual adjustment of stimulus parameters (frequency difference of the tones in audition, opening angle of the gratings in vision) such that integrated and segregated percepts would be equally likely in each participant and modality. For vision, the adjustment procedure produced a balanced distribution of integrated and segregated percepts at group level and avoided strong individual asymmetries. In contrast, the adjustment was not entirely successful for auditory multistability, and thus also not for equating the proportion of integrated and segregated percepts across modalities. Although we were able to account for this in our analysis, future studies should improve the auditory parameter adjustment. The adjustment part most likely needs to be prolonged to allow for multistability to stabilize (Denham et al., 2014). Yet importantly, the individual adjustment successfully abolished any correlations between the proportions of integrated/segregated percepts in the visual and auditory modalities across participants. This rules out that participants who show consistent asymmetries in both modalities dominate the overall measure of coupling (though such asymmetries could also be accounted for in the analysis; see Hupé et al., 2008). The current outcome that visual perception was symmetric while auditory perception tended to be asymmetric in many participants, in fact *reduced* the chance of finding bimodal coupling: if one of the percepts is more frequent in one modality than the other, the overall consistency cannot be 100%. The observed values (proportion of integrated/segregated within each modality) give a theoretical maximum of the consistency that can be reached. This—in addition with limitations in auditory report quality and in the accuracy of visual decoding—put an upper limit on the moment-by-moment coupling that could be obtained (i.e., the expectable maximum was far below 100%). It is remarkable that above-chance coupling was nevertheless observed at group level. Future studies should mainly improve on the symmetry of auditory percept distributions (via suitable parameter choices) and on the quality of auditory report, both of which can be achieved by a longer adjustment and training procedure (Denham et al., 2014; Einhäuser, Thomassen, & Bendixen, 2017). Here, we deliberately invited participants with little to no experience in such paradigms to avoid effects of excessive training. We predict that with improvements in terms of a controlled amount of unimodal training, even higher degrees of bimodal coupling will be observable, which will be beneficial for studying the involved mechanisms in more detail (such as whether one modality is “driving” the other in terms of consistent perceptual switching).

The degree of bimodal coupling observed herein is highly similar to the “common time” of corresponding percepts found by Hupé et al. (2008) for their combination of auditory streaming and pattern-component rivalry (see their Figure 5A, “plaid” condition). The current study transfers this from a dual-task situation (Hupé et al., 2008) to a measurement without response interference, which rules out alternative explanations at post-perceptual stages and suggests that indeed perceptual effects underlie the co-occurrence of corresponding percepts. It is tempting to speculate on the mechanisms of this bimodal coupling. Although there are corresponding perceptual interpretations (integrated/segregated), the auditory and visual stimuli were not so similar to each other as to involve strong perceptual grouping effects. In particular, the motions of the plaid or gratings were not in any way coupled to the timescales of the sounds (unlike for instance in Einhäuser et al., 2017, where we explicitly induced such temporal coupling). We therefore consider it unlikely that the two stimuli were bound into one bimodal perceptual object, as if the plaid were emitting the ABA_ tone sequence and the gratings were emitting the separate A and B tone sequences. Previous studies explicitly inducing perceptual grouping (e.g., Conrad et al., 2012; the same-orientation condition of Adams & Haire, 1959; the “motion” condition of Hupé et al., 2008) found a much higher degree of co-occurrence between the percepts. Here, we deliberately use percepts that are compatible (one object/two objects in each modality), but do not group together as a bimodal object. That we nevertheless observe bimodal coupling indicates a more global tendency to perceive corresponding percepts when presented with stimuli evoking multistability in two modalities simultaneously. This global tendency could be instantiated neurally by top-down mechanisms such as a central oscillator (Carter & Pettigrew, 2003) tuning the sensitivity of modality-specific neurons for a particular percept. Alternatively, it could arise in a bottom-up manner: For the visual modality, long-range lateral coupling between neurons of similar tuning has been suggested as a possible neural mechanism to facilitate a common perceptual interpretation of two visual figures (Klink, Noest, Holten, van den Berg, & van Wezel, 2009). This concept could be extended toward the integration of distinct components into a coherent pattern or object, which is fundamental in visual and auditory scene analysis alike. Thus the bimodal coupling effects observed herein could be explained by bottom-up or top-down integration, and are generally in line with models postulating a combination of local and global mechanisms to account for multistable perception (Carter & Pettigrew, 2003; Klink et al., 2008; Long & Toppino, 2004; Noest, van Ee, Nijs, & van Wezel, 2007; Sterzer et al., 2009; Walker, 1978).

**Figure 5.**
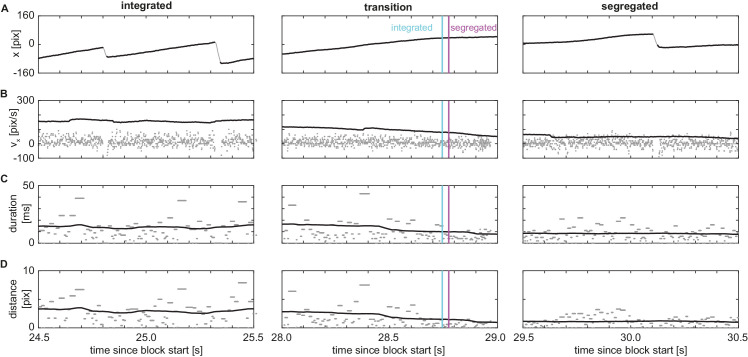
Zoomed-in sections of the data of Figure 2 in the main text with individual samples represented as dots. Vertical panels (**A**–**D**) correspond to those of Figure 2. From left to right: one-second excerpts from representative periods in which integration was reported (left), a transition occurred from integrated to segregated report (middle, cyan line: end of integrated report, magenta line: start of segregated report), and a period with segregated report (right). Time axis on bottom applies to all panels, and times correspond to those of Figure 2. Note how periods of integration have steeper slow-phase slopes (i.e., higher horizontal velocities in the OKN slow phase direction; A), which correspond to a higher average speed (B; note that the raw data is scaled down as in Figure 2B), than periods of segregation. Moreover, fast phases are longer in duration (**C**) and extent (**D**) during integration than during segregation. Also note how the measures in panels **C** and **D** are insensitive to noise in the velocity estimates, because short detections of velocities in the fast-phase direction (isolated bits with low duration and extent) contribute little to the sliding average.

An interesting future avenue for refining putative mechanisms would be to investigate whether the bimodal coupling is uni- or bidirectional. Although we clearly demonstrate moment-by-moment coupling (i.e., co-occurrence of corresponding percepts), we do not investigate any causal effects, that is, whether the perceptual switches in both modalities happen at the same time, and if so, which modality takes the lead in such switches. Future studies can assess more specifically whether an induced perceptual change in one modality directly leads to a re-interpretation in the other modality, using the stimuli and methods employed herein. This will link our interference-free measurement even more closely with previous studies studying similar questions in a dual-task setting (Hupé et al., 2008).

In conclusion, we have reported evidence for bimodal moment-by-moment coupling in perceptual multistability. The coupling is not perfect: a considerable degree of independence in perceptual decisions across modalities remains. This is consistent with the results of previous studies investigating such coupling in dual-task settings. Relative to those previous studies, our measurement approach rules out response interference as a possible confounding factor. The current results are well in line with models postulating a combination of local and global mechanisms to account for multistable perception (Carter & Pettigrew, 2003; Klink et al., 2008; Long & Toppino, 2004; Noest et al., 2007; Sterzer et al., 2009; Walker, 1978).

## Supplementary Material

Supplement 1

Supplement 2

Supplement 3

Supplement 4

## Footnotes

1 In principle, the same model can be selected twice, if both decoded visual unimodal blocks yield the same optimal cost parameter. This is the case in a total of eight cases (out of 48 [16 × 3] pairs of models trained on the same block). Because this model should not be down-weighted, this is not corrected in the analysis (i.e. the model is used twice, ignoring the redundancy). Because there is no independence assumption when using the selected models, this is uncritical for statistical analysis.

## Appendix Appendix

As input to the SVM classifier, we extracted the time courses of three variables from the raw horizontal eye-position data after separating fast and slow phases of the OKN (Figures 2A, 5A): a smoothed slow-phase velocity (Figures 2B, 5B), a robust measure of the duration of OKN fast phases (Figures 2C, 5C) and of the distance covered by the fast phases (Figures 2D, 5D). As a first step, periods of blinks, as detected by the Eyelink software, were marked as missing data for all variables. For technical reasons, the start and end of a blink event are surrounded by the start and the end of an apparent saccade; for the purpose of analysis, the apparent saccade's start and end were used as the start and end of the blink.

○ To characterize the OKN slow phase, we measure the horizontal velocity component of the eye-movement data. For analyzing this variable (*horizontal velocity*), we treated all detected saccades (using the Eyelink software with thresholds of 35°/s for velocity and 9500°/s^2^ for acceleration) as missing data, as they typically correspond to OKN fast phases (Figure 5A). We then determined the horizontal velocity at any given timepoint as the difference in position between this timepoint and the next (Figure 5B, gray). We averaged these differences in an interval of ±500 ms around each timepoint (1001 datapoints, Figure 5B, black), where missing data were ignored (i.e., if there are *k* missing values, the average is taken over 1001 − *k* datapoints).
○ As second variable, we used the duration of consecutive data points in the direction opposing the plaid drift direction (i.e., going from right to left). Including saccades but excluding blinks (and surrounding apparent saccades), we identified the direction between subsequent timepoints (left or right). We then identified chunks of timepoints with velocity to the left and assigned the duration of the chunk to each datapoint in this chunk. For example, if the eye moved leftward for 50 ms, all the points between start and end of this period got assigned a value of 50 ms (Figure 5C, gray). This approach benefits from the low noise levels of the recording and characterizes fast phase durations. The trace of durations was then averaged as above (Figure 5C, black), treating blinks as missing data.
○ As third variable we used the leftward chunks as for the second variable, and measured the horizontal distance covered within each chunk (Figure 5D, gray), again averaged with a one-second (±500 ms) window (Figure 5D, black). This measure relates to the spatial length of OKN fast phases.

This procedure results in three variables as function of time. For each timepoint, each of these three variables was sampled at 21 timepoints relative to the current one by shifting in 100-ms steps (i.e., at −2.0 seconds, −1.9 seconds, …−0.2 seconds, −0.1 seconds, and 0 seconds relative to the analyzed timepoint) yielding 63 features (21 × 3) per timepoint. Consequently, for all timepoints, we obtained a 63-dimensional feature vector that characterized eye movements in a time window from −2.5 seconds to 0.5 second (two seconds of samples, one second averaging window) relative to the timepoint of consideration, as well as a label (the reported percept). Timepoints for which any feature or the label was missing (because neither of the two percepts was exclusively reported) were not included in training or testing the SVM. For training the SVM, we subsampled this training data at 100 Hz for computational efficiency^2^; for testing the SVM, the full 1 kHz sampling was used.

## Supplementary Material

**Supplementary Movie S1.** Simultaneous presentation of auditory streaming stimulus and visual pattern-component rivalry stimulus (bimodal condition evoking multistable perception).

**Supplementary Movie S2.** Disambiguated version of the visual stimulus, alternating between suggesting an integrated and a segregated percept (twice each for about 7.5 s).

**Supplementary Soundfile S3.** Disambiguated version of the auditory stimulus, alternating between suggesting an integrated and a segregated percept (twice each for about 7.5 s).
