# Supplementary material for: Bimodal moment-by-moment coupling in perceptual multistability
Source: J Vis. 2024 May 31;24(5):16. doi: 10.1167/jov.24.5.16 (PMC11146044; doi:10.1167/jov.24.5.16)
Supplement: Supplement 1 [file jovi-24-5-16_s001.pdf]

Bimodal moment-by-moment coupling in perceptual multistability

Supplementary Material: Derivation of Theoretical and Expected Bounds on Consistency

Grenzebach, J., Wegner, T. G. G., Einhäuser, W., & Bendixen, A. (*Journal of Vision*, 2024)

In this supplementary material, we derive the upper and lower bounds on the consistency measure for each participant.

The *theoretical* bounds (theoretical maximum and minimum) take into account the asymmetry between segregated and integrated percepts within each modality as well as the differences between the proportion of segregated / integrated percepts between the modalities. This means, they are dependent on the proportion of auditory integrated percepts ($p_{aud=int}$) and visual integrated percepts ($p_{vis=int}$). The reverse proportions of auditory segregated percepts ($p_{aud=seg}$) and visual segregated percepts ($p_{vis=seg}$) are then fixed, as $p_{aud=int} + p_{aud=seg} = 1$ and likewise $p_{vis=int} + p_{vis=seg} = 1$, such that there are only two free values. These values are measured for the multistable part of each bimodal block separately and - in case of p_{vis}, which is decoded using the SVM models - separately for each model, and then averaged across blocks and models.[1] The theoretical bounds are hard, in the sense that they cannot be exceeded by empirical data.

The *expected bounds* (expected maximum and expected minimum) in addition take into account that neither decoding nor report is perfect. For each participant, we refer to the probability of correctly reporting the auditory percept as r. For each participant and SVM model, we refer to the probability of correctly decoding the visual percept as d. We estimate r as the average accuracy from the catch phases of the bimodal blocks of the main experimental session and d as the class-adjusted accuracy of the respective model in the unimodal visual blocks. The expected bounds, which each depend on four values $p_{aud=int}$, $p_{vis=int}$, r and d, are probabilistic bounds, i.e. they can be exceeded if decoding or report happens to be better or worse in the respective condition than estimated from blocks and parts with available ground truth.

The theoretical bounds can be retrieved as a special case of the expected bounds by setting $r = d = 1$. Hence, in the following, we derive the expected bounds first and then set $r = d = 1$ to retrieve the theoretical bounds.

Expected maximum consistency

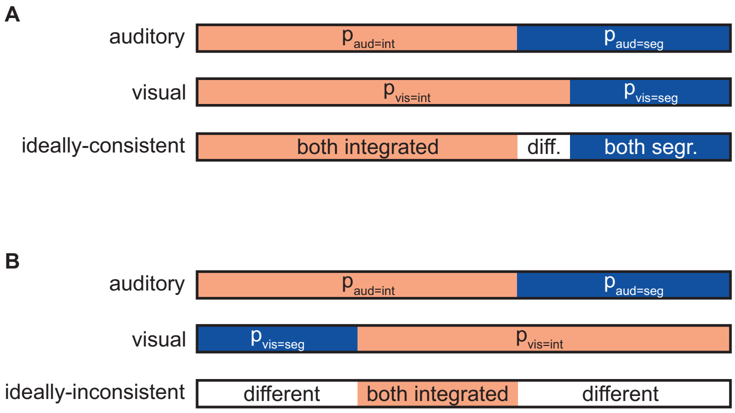

Figure S1: **Theoretical bounds on coupling given different proportions of integrated/segregated percepts across modalities.** A) Participant with "ideal" consistency (theoretical upper bound), illustrated for an example with $p_{vis=int} > p_{aud=int}$. B) "Ideally-inconsistent" participant (theoretical lower bound), same example as in panel A.

[1]Theoretically, aggregating across blocks is also possible, but does not yield qualitatively different results in practice.

The logic of the derivation follows the notion of an ideal observer or ideal listener model in vision and audition, respectively. We consider a participant that is "ideally consistent", that is, has the same percept in vision and audition whenever possible. Figure S1A illustrates for a case where $p_{vis=int} > p_{aud=int}$ that the consistency this "ideally-consistent" participant can reach is not 100%: If the participant perceives visual integration at each timepoint they also perceive auditory integration (left part of the Figure S1A), and if they perceive auditory segregation at each timepoint they also perceive visual segregation (right part of Figure S1A), there remains a "gap". For an "ideally-consistent" participant, the maximum proportion with both percepts integrated is given by the minimum of $p_{vis=int}$ and $p_{aud=int}$:

$$\text{“Ideal” proportion of both percepts integrated: } \min(p_{vis=int}, p_{aud=int}).$$

Conversely the maximum proportion with both percepts segregated is given by the minimum of $p_{vis=seg}$ and $p_{aud=seg}$:

$$\text{“Ideal” proportion of both percepts segregated: } \min(p_{vis=seg}, p_{aud=seg}).$$

Since

$$\begin{aligned}&\min(p_{vis=seg}, p_{aud=seg}) =\\&\min(1 - p_{vis=int}, 1 - p_{aud=int}) =\\&1 - \max(p_{vis=int}, p_{aud=int})\end{aligned}$$

we only have to consider the cases $p_{vis=int} > p_{aud=int}$ and $p_{vis=int} < p_{aud=int}$ separately.

In the case ($p_{vis=int} > p_{aud=int}$), an "ideally-consistent" participant has three different combinations of percepts with the following proportions (Figure S1A):

- integrated percepts in both modalities: $p_{aud=int}$
- segregated percepts in both modalities: $p_{vis=seg} = 1 - p_{vis=int}$
- auditory segregated, visual integrated percept:
  $1 - (p_{aud=int} + 1 - p_{vis=int}) = p_{vis=int} - p_{aud=int}$

In the case ($p_{vis=int} < p_{aud=int}$), the roles of integration and segregation are swapped, and one obtains:

- integrated percepts in both modalities: $p_{vis=int}$
- segregated percepts in both modalities: $p_{aud=seg} = 1 - p_{aud=int}$
- auditory integrated, visual segregated percept:
  $1 - (p_{vis=int} + 1 - p_{aud=int}) = p_{aud=int} - p_{vis=int}$

In the case ($p_{vis=int} = p_{aud=int}$), the ideally-consistent participant achieves 100% consistency:

- integrated percepts in both modalities: $p_{aud=int} = p_{vis=int}$
- segregated percepts in both modalities: $p_{aud=seg} = p_{vis=seg}$

From these proportions, we can compute the theoretical maximum consistency for each participant. For the expected maximum consistency, however, we have to appreciate that we do not have access to the true percept, but only to the participant's report (auditory percept) and decoding result (visual percept), respectively. This implies that four cases have to be distinguished:

- auditory percept is reported correctly, visual percept is decoded correctly
- auditory percept is reported correctly, visual percept is decoded incorrectly
- auditory percept is reported incorrectly, visual percept is decoded correctly
- auditory percept is reported incorrectly, visual percept is decoded incorrectly

With the abbreviations r for reporting accuracy and d for decoding accuracy as introduced above, these cases have the proportions (under the reasonable assumption that recoding errors and decoding errors at any point in time are independent of each other):

- both modalities correct: $r \times d$
- auditory correct, visual incorrect: $r \times (1 - d)$
- auditory incorrect, visual correct: $(1 - r) \times d$
- both modalities incorrect: $(1 - r) \times (1 - d)$

Since we compute the overall consistency of the percepts as class-adjusted decoding accuracy in the bimodal blocks, using the decoded visual percept as "predicted class" in the SVM sense and the reported auditory percept as "true class" (label), the expected and theoretical maximum consistencies have to use the same calculation approach. That is, we need to compute the class-adjusted accuracy for an ideally-consistent participant with imperfect reporting and decoding. Specifically, for the ideally-consistent participant, we have to compute

$$accuracy_{segregated} = \frac{\#\text{timepoints model predicts segregated visual percept and segregated auditory percept is reported}}{\#\text{timepoints segregated auditory percept is reported}}$$

$$accuracy_{integrated} = \frac{\#\text{timepoints model predicts integrated visual percept and integrated auditory percept is reported}}{\#\text{timepoints integrated auditory percept is reported}}$$

and then average these two values.

For each of the three possible percept combinations - both modalities integrated, both modalities segregated, modalities different - we have to consider the four different outcomes of reporting/decoding. All three combinations in which the reported auditory percept is segregated and the decoded visual percept is also segregated will then add up to the nominator of $accuracy_{segregated}$; all six combinations in which the reported auditory percept is segregated (irrespective of the decoded visual percept) will add up to the denominator of $accuracy_{segregated}$, and so on. The twelve combinations and their contribution to the $accuracy_{segregated}$ and $accuracy_{integrated}$ are tabulated in Table S1 for the case $p_{vis=int} > p_{aud=int}$ and in Table S2 for the case $p_{vis=int} < p_{aud=int}$.

true percept	proportion of true percept	auditory reported	visual decoded	proportion of measured (reported/decoded) percept	contributes to (summand) acc_{seg} nom.	acc_{seg} denom.	acc_{int} nom.	acc_{int} denom.
both integrated	$p_{aud=int}$	integrated	integrated	$p_{aud=int} \times r \times d$			X	X
		segregated	integrated	$p_{aud=int} \times (1-r) \times d$		X		
		integrated	segregated	$p_{aud=int} \times r \times (1-d)$				X
		segregated	segregated	$p_{aud=int} \times (1-r) \times (1-d)$	X	X		
both segregated	$1 - p_{vis=int}$	integrated	integrated	$(1-p_{vis=int}) \times (1-r) \times (1-d)$			X	X
		segregated	integrated	$(1-p_{vis=int}) \times r \times (1-d)$		X		
		integrated	segregated	$(1-p_{vis=int}) \times (1-r) \times d$				X
		segregated	segregated	$(1-p_{vis=int}) \times r \times d$	X	X		
auditory segregated visual integrated	$p_{vis=int} - p_{aud=int}$	integrated	integrated	$(p_{vis=int} - p_{aud=int}) \times (1-r) \times d$			X	X
		segregated	integrated	$(p_{vis=int} - p_{aud=int}) \times r \times d$		X		
		integrated	segregated	$(p_{vis=int} - p_{aud=int}) \times (1-r) \times (1-d)$				X
		segregated	segregated	$(p_{vis=int} - p_{aud=int}) \times r \times (1-d)$	X	X		

Table S1: **Ideally-consistent participant with $\mathbf{p_{vis=int} > p_{aud=int}}$.** True percepts assume ideally-consistent participant; proportions of true percepts as given in text; green background highlights correct report/decoding; proportion of measured percepts combine the proportion of the true percept with the probability to report/decode correctly (r,d) or incorrectly ($1-r$, $1-d$); final columns denote contribution to accuracy formula (nom.=nominator; denom.=denominator), this corresponds to the "auditory reported" (contributes to nominator and denominator) and "visual decoded" (contributes to nominator only) column for segregated and integrated accuracy, respectively.

true percept	proportion of true percept	auditory reported	visual decoded	proportion of measured (reported/decoded) percept	contributes to (summand) acc_{seg} nom.	acc_{seg} denom.	acc_{int} nom.	acc_{int} denom.
both integrated	$p_{vis=int}$	integrated	integrated	$p_{vis=int} \times r \times d$			X	X
		segregated	integrated	$p_{vis=int} \times (1-r) \times d$		X		
		integrated	segregated	$p_{vis=int} \times r \times (1-d)$				X
		segregated	segregated	$p_{vis=int} \times (1-r) \times (1-d)$	X	X		
both segregated	$1 - p_{aud=int}$	integrated	integrated	$(1-p_{aud=int}) \times (1-r) \times (1-d)$			X	X
		segregated	integrated	$(1-p_{aud=int}) \times r \times (1-d)$		X		
		integrated	segregated	$(1-p_{aud=int}) \times (1-r) \times d$				X
		segregated	segregated	$(1-p_{aud=int}) \times r \times d$	X	X		
auditory integrated visual segregated	$p_{aud=int} - p_{vis=int}$	integrated	integrated	$(p_{aud=int} - p_{vis=int}) \times r \times (1-d)$			X	X
		segregated	integrated	$(p_{aud=int} - p_{vis=int}) \times (1-r) \times (1-d)$		X		
		integrated	segregated	$(p_{aud=int} - p_{vis=int}) \times r \times d$				X
		segregated	segregated	$(p_{aud=int} - p_{vis=int}) \times (1-r) \times d$	X	X		

Table S2: **Ideally-consistent participant with $\mathbf{p_{vis=int} < p_{aud=int}}$.** Notation as in Table S1. Note that for the first eight rows, only the true proportions change relative to Table S1, while in the final four rows also the roles of integration and segregation (and therefore of r and $1-r$ as well as of d and $1-d$) swap.

For the case $p_{vis=int} > p_{aud=int}$, we collect the terms according to Table S1 and obtain:

$$accuracy_{segregated} = \frac{p_{aud=int}(1-r)(1-d)+(1-p_{vis=int})rd+(p_{vis=int}-p_{aud=int})r(1-d)}{p_{aud=int}(1-r)d+p_{aud=int}(1-r)(1-d)+(1-p_{vis=int})r(1-d)+(1-p_{vis=int})rd+(p_{vis=int}-p_{aud=int})rd+(p_{vis=int}-p_{aud=int})r(1-d)}$$

and

$$accuracy_{integrated} = \frac{p_{aud=int}rd+(1-p_{vis=int})(1-r)(1-d)+(p_{vis=int}-p_{aud=int})(1-r)d}{p_{aud=int}rd+p_{aud=int}r(1-d)+(1-p_{vis=int})(1-r)(1-d)+(1-p_{vis=int})(1-r)d+(p_{vis=int}-p_{aud=int})(1-r)d+(p_{vis=int}-p_{aud=int})(1-r)(1-d)}$$

.

This simplifies to

$$accuracy_{segregated} = \frac{rd + (1 + 2rd - 2r - d)p_{aud=int} + (r - 2rd)p_{vis=int}}{r + (1 - 2r)p_{aud=int}}$$

and

$$accuracy_{integrated} = \frac{1 - r - d + rd + (2rd - d)p_{aud=int} + (r - 1 - 2rd + 2d)p_{vis=int}}{1 - r + (2r - 1)p_{aud=int}}$$

, respectively.

Analogously, for the case $p_{vis=int} < p_{aud=int}$, we collect the summands from Table S2 and obtain after simplification:

$$accuracy_{segregated} = \frac{rd + (d - 2rd)p_{aud=int} + (2rd - 2d - r + 1)p_{vis=int}}{r + (1 - 2r)p_{aud=int}}$$

and

$$accuracy_{integrated} = \frac{1 - r - d + rd + (-2rd + 2r + d - 1)p_{aud=int} + (2rd - r)p_{vis=int}}{1 - r + (2r - 1)p_{aud=int}}$$

.

In the boundary case of $p_{vis=int} = p_{aud=int}$, we obtain:

$$accuracy_{segregated} = \frac{rd + (1 - d - r)p_{aud=int}}{r + (1 - 2r)p_{aud=int}}$$

and

$$accuracy_{integrated} = \frac{1 - r - d + rd + (r + d - 1)p_{aud=int}}{1 - r + (2r - 1)p_{aud=int}}$$

.

In each of the cases, and analogously to the computation for the empirical data, the mean of the two terms is the *expected maximum consistency.*

Theoretical maximum consistency

The theoretical maximum consistency is retrieved by setting $r = d = 1$ in the formulae above. For $p_{vis=int} > p_{aud=int}$, we obtain:

$$accuracy_{segregated} = \frac{1 - p_{vis=int}}{1 - p_{aud=int}} = \frac{p_{vis=seg}}{p_{aud=seg}}$$

and

$$accuracy_{integrated} = 1$$

.

For $p_{vis=int} < p_{aud=int}$, we obtain:

$$accuracy_{segregated} = 1$$

and

$$accuracy_{integrated} = \frac{p_{vis=int}}{p_{aud=int}}$$

.

In the case $p_{vis=int} = p_{aud=int}$ both accuracies simplify to 1. The results can be intuitively understood, noting that in the first case, each timepoint with auditory integration can be assigned a timepoint of visual integration (Figure S1A), while for the second case each timepoint with auditory segregation can be assigned a timepoint of visual segregation, but the reverse does not apply in each case. As the consistency is defined as decoding accuracy with the auditory report as the "true label", consistency can only be perfect (in the case of perfect report and decoding) for the class for which each auditory "label" can find a visual "prediction", which is the case for integration if $p_{vis=int} > p_{aud=int}$, for segregation if $p_{vis=int} < p_{aud=int}$, and for both only if $p_{vis=int} = p_{aud=int}$.

Expected minimum consistency

Similar to the theoretical maximum consistency being below 100%, the theoretical minimum is above 0%. We first consider a case in which $p_{vis=int} + p_{aud=int} > 1$ (which is equivalent to $p_{vis=seg} + p_{aud=int} < 1$, $p_{vis=int} > p_{aud=seg}$, and $p_{vis=seg} < p_{aud=int}$, as can be seen by using $p_{aud=int} = 1 - p_{aud=seg}$ and $p_{vis=int} = 1 - p_{vis=seg}$). As illustrated in Figure S1B, a participant whose consistency is as small as possible ("ideally *in*consistent") can achieve distinct percepts for a proportion of $p_{vis=seg} + p_{aud=seg}$, but necessarily has periods for which both percepts are identical (integrated in the case of $p_{vis=int} + p_{aud=int} > 1$). That is, for the case ($p_{vis=int} + p_{aud=int} > 1$), this "ideally-inconsistent" participant has three different combinations of percepts with the following proportions (FigureS1B):

- auditory segregated, visual integrated percept for $p_{aud=seg} = 1 - p_{aud=int}$
- auditory integrated, visual segregated percept for $p_{vis=seg} = 1 - p_{vis=int}$
- integrated percepts in both modalities for $1 - (p_{vis=seg} + p_{aud=seg}) = p_{vis=int} + p_{aud=int} - 1$

Conversely for the case $p_{vis=int} + p_{aud=int} < 1$, we obtain:

- auditory segregated, visual integrated percept for $p_{vis=int}$
- auditory integrated, visual segregated percept for $p_{aud=int}$
- segregated percepts in both modalities for $1 - (p_{vis=int} + p_{aud=int})$

For the boundary case of $p_{vis=int} + p_{aud=int} = 1$, a condition equivalent to $p_{vis=int} = p_{aud=seg}$ and $p_{vis=seg} = p_{aud=int}$, the "ideally-inconsistent" participant will reach 0 consistency with

- auditory segregated, visual integrated percept for $p_{aud=seg} = p_{vis=int}$
- auditory integrated, visual segregated percept for $p_{aud=int} = p_{vis=seg}$

As for the ideally-consistent participant, we have to take into account the decoding and reporting accuracies, since decoding and reporting errors in the case of the ideally-inconsistent participant increase consistency. We follow the same procedure as in the ideally-consistent case. Table S3 and Table S4 contain the resulting contributions to the expression for $accuracy_{segregated}$ and $accuracy_{integrated}$, Table S3 for the case $p_{vis=int} + p_{aud=int} > 1$, Table S4 for the case $p_{vis=int} + p_{aud=int} < 1$.

true percept	proportion of true percept	auditory reported	visual decoded	proportion of measured (reported/decoded) percept	contributes to (summand) acc_{seg} nom.	acc_{seg} denom.	acc_{int} nom.	acc_{int} denom.
both integrated	$p_{vis=int} + p_{aud=int} - 1$	integrated	integrated	$(p_{vis=int} + p_{aud=int} - 1) \times r \times d$			X	X
		segregated	integrated	$(p_{vis=int} + p_{aud=int} - 1) \times (1 - r) \times d$		X		
		integrated	segregated	$(p_{vis=int} + p_{aud=int} - 1) \times r \times (1 - d)$				X
		segregated	segregated	$(p_{vis=int} + p_{aud=int} - 1) \times (1 - r) \times (1 - d)$	X	X		
auditory segregated & visual integrated	$1 - p_{aud=int}$	integrated	integrated	$(1 - p_{aud=int}) \times (1 - r) \times d$			X	X
		segregated	integrated	$(1 - p_{aud=int}) \times r \times d$		X		
		integrated	segregated	$(1 - p_{aud=int}) \times (1 - r) \times (1 - d)$				X
		segregated	segregated	$(1 - p_{aud=int}) \times r \times (1 - d)$	X	X		
auditory integrated & visual segregated	$1 - p_{vis=int}$	integrated	integrated	$(1 - p_{vis=int}) \times r \times (1 - d)$			X	X
		segregated	integrated	$(1 - p_{vis=int}) \times (1 - r) \times (1 - d)$		X		
		integrated	segregated	$(1 - p_{vis=int}) \times r \times d$				X
		segregated	segregated	$(1 - p_{vis=int}) \times (1 - r) \times d$	X	X		

Table S3: **Ideally-inconsistent participant with $\mathbf{p_{vis=int} + p_{aud=int} > 1}$.** True percepts assume ideally-inconsistent participant; notation as in Tables S1 and S2

true percept	proportion of true percept	auditory reported	visual decoded	proportion of measured (reported/decoded) percept	contributes to (summand) acc_{seg} nom.	acc_{seg} denom.	acc_{int} nom.	acc_{int} denom.
both segregated	$1 - p_{vis=int} - p_{aud=int}$	integrated	integrated	$(1 - p_{vis=int} - p_{aud=int}) \times (1 - r) \times (1 - d)$			X	X
		segregated	integrated	$(1 - p_{vis=int} - p_{aud=int}) \times r \times (1 - d)$		X		
		integrated	segregated	$(1 - p_{vis=int} - p_{aud=int}) \times (1 - r) \times d$				X
		segregated	segregated	$(1 - p_{vis=int} - p_{aud=int}) \times r \times d$	X	X		
auditory segregated & visual integrated	$p_{vis=int}$	integrated	integrated	$(p_{vis=int}) \times (1 - r) \times d$			X	X
		segregated	integrated	$(p_{vis=int}) \times r \times d$		X		
		integrated	segregated	$(p_{vis=int}) \times (1 - r) \times (1 - d)$				X
		segregated	segregated	$(p_{vis=int}) \times r \times (1 - d)$	X	X		
auditory integrated & visual segregated	$p_{aud=int}$	integrated	integrated	$(p_{aud=int}) \times r \times (1 - d)$			X	X
		segregated	integrated	$(p_{aud=int}) \times (1 - r) \times (1 - d)$		X		
		integrated	segregated	$(p_{aud=int}) \times r \times d$				X
		segregated	segregated	$(p_{aud=int}) \times (1 - r) \times d$	X	X		

Table S4: **Ideally-inconsistent participant with $\mathbf{p_{vis=int} + p_{aud=int} < 1}$.** True percepts assume ideally-inconsistent participant; notation as in Tables S1 through S3

For the case $p_{vis=int} + p_{aud=int} > 1$, we collect the terms according to Table S3, simplify the resulting expressions and obtain:

$$accuracy_{segregated} = \frac{2r + 2d - 3rd - 1 + (2rd - 2r - d + 1)p_{aud=int} + (2rd - 2d - r + 1)p_{vis=int}}{r + (1 - 2r)p_{aud=int}}$$

and

$$accuracy_{integrated} = \frac{r + d - 3rd + (2rd - d)p_{aud=int} + (2rd - r)p_{vis=int}}{1 - r + (2r - 1)p_{aud=int}}$$

.
For the case $p_{vis=int} + p_{aud=int} < 1$, we collect the terms according to Table S4, simplify the resulting expressions and obtain:

$$accuracy_{segregated} = \frac{rd + (d - 2rd)p_{aud=int} + (r - 2rd)p_{vis=int}}{r + (1 - 2r)p_{aud=int}}$$

and

$$accuracy_{integrated} = \frac{1 - r - d + rd + (d - 1 - 2rd + 2r)p_{aud=int} + (r - 1 - 2rd + 2d)p_{vis=int}}{1 - r + (2r - 1)p_{aud=int}}$$

.
For the case $p_{vis=int} + p_{aud=int} = 1$, there are no phases of identical percepts, and the expressions simplify to

$$accuracy_{segregated} = \frac{r - rd + (d - r)p_{aud=int}}{r + (1 - 2r)p_{aud=int}}$$

and

$$accuracy_{integrated} = \frac{d - rd + (r - d)p_{aud=int}}{1 - r + (2r - 1)p_{aud=int}}$$

.
In all cases, the expected minimum consistency is obtained by averaging the terms for $accuracy_{segregated}$ and $accuracy_{integrated}$.

Theoretical minimum consistency

We retrieve the theoretical minimum consistency by setting $r = d = 1$ in the formulae above and obtain for $p_{vis=int} + p_{aud=int} > 1$

$$accuracy_{segregated} = 0$$

and

$$accuracy_{integrated} = \frac{p_{aud=int} + p_{vis=int} - 1}{p_{aud=int}}$$

For the case $p_{vis=int} + p_{aud=int} < 1$, we obtain

$$accuracy_{segregated} = \frac{1 - (p_{aud=int} + p_{vis=int})}{1 - p_{aud=int}} = \frac{p_{aud=seg} - p_{vis=int}}{p_{aud=seg}}$$

$$accuracy_{integrated} = 0$$

For the boundary case $p_{vis=int} + p_{aud=int} = 1$ both accuracies and thus the theoretical minimum are 0 in case of perfect report and decoding.

The thus obtained theoretical and expected upper and lower bounds on the consistency measure for each participant are displayed in Figure 4 of the main text for descriptive comparison to the empirical consistency values. We note that upper and lower bound are not symmetrical around 50% in each case, due to asymmetric contributions from the values of r, d, $p_{vis=int}$, and $p_{aud=int}$. Nevertheless, chance level for statistical comparison is at 50% in each case.

Note on the chance level of 50%

The main reason to choose the consistency measure as a class-adjusted accuracy is avoiding biases due to one class having a higher proportion than the other (thereby dominating the overall score). As a consequence, chance level is at 50%, irrespective of the values of r, d, $p_{vis=int}$, and $p_{aud=int}$. This can also be seen when considering a participant that shows random consistency for any given set of these four variables. A participant that shows random consistency will have:

- segregated percepts in both modalities: $p_{aud=seg} \times p_{vis=seg} = (1 - p_{aud=int}) \times (1 - p_{vis=int})$
- auditory integrated, visual segregated percept for $p_{aud=int} \times (1 - p_{vis=int})$
- auditory segregated, visual integrated percept for $(1 - p_{aud=int}) \times p_{vis=int}$
- integrated percepts in both modalities: $p_{aud=int} \times p_{vis=int}$

There is no need to differentiate cases for these formulae, so we obtain a table with 16 (4 × 4) combinations for computing the expected consistency for a participant who couples the modalities at random (Table S5).

true percept	proportion of true percept	auditory reported	visual decoded	proportion of measured (reported/decoded) percept	contributes to (summand) acc_{seg} nom.	acc_{seg} denom.	acc_{int} nom.	acc_{int} denom.
both segregated	$(1 - p_{aud=int}) \times (1 - p_{vis=int})$	integrated	integrated	$(1 - p_{aud=int}) \times (1 - p_{vis=int}) \times (1 - r) \times (1 - d)$			X	X
		segregated	integrated	$(1 - p_{aud=int}) \times (1 - p_{vis=int}) \times r \times (1 - d)$		X		
		integrated	segregated	$(1 - p_{aud=int}) \times (1 - p_{vis=int}) \times (1 - r) \times d$				X
		segregated	segregated	$(1 - p_{aud=int}) \times (1 - p_{vis=int}) \times r \times d$	X	X		
auditory segregated & visual integrated	$(1 - p_{aud=int}) \times p_{vis=int}$	integrated	integrated	$(1 - p_{aud=int}) \times p_{vis=int} \times (1 - r) \times d$			X	X
		segregated	integrated	$(1 - p_{aud=int}) \times p_{vis=int} \times r \times d$		X		
		integrated	segregated	$(1 - p_{aud=int}) \times p_{vis=int} \times (1 - r) \times (1 - d)$				X
		segregated	segregated	$(1 - p_{aud=int}) \times p_{vis=int} \times r \times (1 - d)$	X	X		
auditory integrated & visual segregated	$p_{aud=int} \times (1 - p_{vis=int})$	integrated	integrated	$p_{aud=int} \times (1 - p_{vis=int}) \times r \times (1 - d)$			X	X
		segregated	integrated	$p_{aud=int} \times (1 - p_{vis=int}) \times (1 - r) \times (1 - d)$		X		
		integrated	segregated	$p_{aud=int} \times (1 - p_{vis=int}) \times r \times d$				X
		segregated	segregated	$p_{aud=int} \times (1 - p_{vis=int}) \times (1 - r) \times d$	X	X		
both integrated	$p_{aud=int} \times p_{vis=int}$	integrated	integrated	$p_{aud=int} \times p_{vis=int} \times r \times d$			X	X
		segregated	integrated	$p_{aud=int} \times p_{vis=int} \times (1 - r) \times d$		X		
		integrated	segregated	$p_{aud=int} \times p_{vis=int} \times r \times (1 - d)$				X
		segregated	segregated	$p_{aud=int} \times p_{vis=int} \times (1 - r) \times (1 - d)$	X	X		

Table S5: **Participant with random coupling across modalities.** True percepts assume participant with random consistency between modalities; notation as in Tables S1 through S4.

Collecting the terms of Table S5, one obtains for the individual accuracies:

$$accuracy_{segregated} = d + (1 - 2d)p_{vis=int}$$

and

$$accuracy_{integrated} = 1 - d + (2d - 1)p_{vis=int}$$

.

While both accuracies depend on d and $p_{vis=int}$, their average is 0.5, independent of these values, validating the use of 0.5 as chance level against which the empirical data are compared.
